# Preparation and Adsorption Photocatalytic Properties of PVA/TiO_2_ Colloidal Photonic Crystal Films

**DOI:** 10.3390/gels10080520

**Published:** 2024-08-07

**Authors:** Zhangyi Qian, Menghan Wang, Junling Li, Zhaoran Chu, Wenwei Tang, Cheng Chen

**Affiliations:** 1 Shanghai Key Laboratory of Engineering Materials Application and Evaluation, School of Energy and Materials, Shanghai Polytechnic University, Shanghai 201209, China; 2 Shanghai Thermophysical Properties Big Data Professional Technical Service Platform, Shanghai Engineering Research Center of Advanced Thermal Functional Materials, Shanghai 201209, China; 3 School of Mathematics Physics and Statistics, Shanghai Polytechnic University, Shanghai 201209, China

**Keywords:** PVA, photocatalytic, TiO_2_, colloidal crystal, photonic crystal

## Abstract

Polyvinyl alcohol (PVA)/TiO_2_/colloidal photonic crystal (CPC) films with photocatalytic properties are presented, where TiO_2_ nanoparticles were introduced into the PVA gel network. Such PVA/TiO_2_/CPC films possess three-dimensional periodic structures that are supported with a PVA/TiO_2_ composite gel. The unique structural color of CPCs can indicate the process of material preparation, adsorption, and desorption. The shift of diffraction peaks of CPCs can be more accurately determined using fiber-optic spectroscopy. The effect of the PVA/TiO_2_/CPC catalyst films showed better properties as the degradation of methylene blue (MB) by the PVA/TiO_2_/CPC film catalyst in 4 h was 77~90%, while the degradation of MB by the PVA/TiO_2_ film was 33% in 4 h, indicating that the photonic crystal structure was 2.3~2.7 times more effective than that of the bulk structure.

## 1. Introduction

With the progress of society and the development of industry, the problem of environmental pollution has become more and more serious. Among these, water resources, as one of the basic resources for human survival, are especially valuable in modern society. However, the daily activities of human beings and industrialized operations have led to numerous water pollution problems, especially dye wastewater, which now accounts for a large proportion of industrial wastewater in China and has become one of the important pollutants in the environment [1,2,3,4,5]. Methylene blue (MB) is a common industrial dye with a high dosage and is widely used in industrial production, which can damage water quality and water ecosystems when it enters water bodies and can cause nausea and vomiting if it enters the human body [6]. Conventional treatments of organically polluted wastewater mainly rely on chemical methods (e.g., inputting photocatalysts) and physical adsorption. Although the chemical methods can decompose organic pollutants by photocatalysis, the effective recovery of the subsequent photocatalysts is also a problem. Physical adsorption is the most commonly used method for treating dyestuff wastewater because of its simple operation, good decolorization efficiency, and the absence of by-products. Conventional adsorbents mainly include activated carbon, ores, modified biosorbents, and so on [7].

Hydrogels, a kind of material with three-dimensional network structures, are widely used as an adsorbent due to their transparent and non-toxic properties, high solubility, high water absorption, and good mechanical properties [8,9]. As one of the oldest hydrogels, polyvinyl alcohol (PVA) hydrogels have been widely used as research objects due to their good chemical stability, non-toxicity, good biocompatibility, high solubility, mechanical properties, and ease of processing [10,11,12]. Currently, materials prepared based on PVA hydrogels have been widely studied in the fields of drug carriers, articular cartilage, wound dressings, photocatalysts, tissue engineering, and other smart materials such as self-healing and shape memory materials, supercapacitors, sensors, and so on. With the development of science and technology, smart gel materials are prevalent, and the devices developed by combining such gels with photonic crystals have great potential. However, the current research on materials in this area is still insufficient due to the limitations of the preparation method. On the one hand, the preparation conditions of photonic crystals are harsh. On the other hand, photonic crystals need to be composited with the gel material without destroying their original functions during the process. Efforts on the physical and chemical modification of PVA to prepare a series of PVA-based hydrogels with a range of functionalities by combining it with colloidal photonic crystals (CPC) [13,14,15,16,17,18], the structural color, slow photon effect, and other optical properties of the CPC are utilized to develop functional photonic hydrogel materials. These photonic gel materials can be applied practically in solution detection, temperature response, photocatalysis, and other fields.

In recent decades, more and more articles have reported on the introduction of photocatalytic titanium dioxide (TiO_2_) nanoparticles into hydrogel systems, where the hydrogel acts as a backbone to adsorb organic dyes in wastewater, and TiO_2_ nanoparticles act as photocatalysts to effectively adsorb degraded organic dyes [19,20,21]. These hydrogel photocatalysts are widely used because the photocatalyst nano-TiO_2_ can be easily recovered from water bodies, and the hydrogel as the skeleton material is non-toxic, biocompatible, and green. However, the photocatalyst nano-TiO_2_ has a low absorption of UV light from natural light and also has a series of drawbacks, such as small adsorption, small specific surface area, and low catalytic activity, which greatly limit its practical application [22]. In recent years, some researchers have found that the slow photon effect of photonic crystals can effectively enhance the photocatalytic efficiency of nano-TiO_2_ photocatalysts, mainly because the opal structure of photonic crystals lengthens the propagation distance of light in the material and slows down the speed of photons at the edges of their blocking bands, which increases the light absorption efficiency of the photocatalysts and thus improves the catalytic activity [23].

In this work, we used a colorless, nontoxic, abrasion-resistant, and biodegradable water-soluble organic polymer polyvinyl alcohol (PVA) hydrogel as the adsorbent skeleton. Titanium dioxide (TiO_2_) nanoparticles were used as the photocatalyst combined with CPCs with structural colors and slow photon effects to prepare a novel photonic crystal gel thin-film photocatalyst; we investigated the adsorption of the film photocatalyst on the efficiency of the photocatalytic adsorption of MB dye from wastewater.

## 2. Results and Discussion

### 2.1. Preparation and Characterization of PVA/TiO_2_/CPC Thin Films

**Figure 1:**
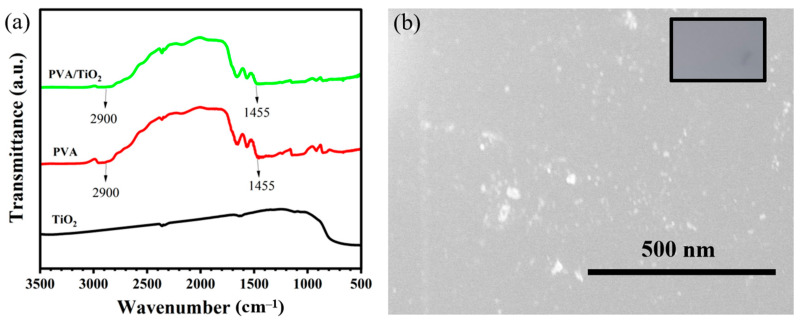
(**a**) Infrared spectra of the PVA, TiO_2_, and mixture of PVA/TiO_2_; (**b**) Scanning electron microscopy (SEM) image of PVA/TiO_2_. The inset is the optical photograph of the sample.

In order to confirm whether there is a new chemical bond formation between TiO_2_ and PVA molecules in the composite films prepared by the simple composite method, the materials were characterized by infrared spectroscopy, and the results are shown in Figure 1. The infrared spectra of our prepared PVA/TiO_2_ and pure PVA samples showed similar profiles, with important characteristic peaks at the same positions, and no new peaks appeared, which was a different phenomenon from that of the PVA/TiO_2_ composite films obtained by high-temperature heat treatment. During the high-temperature heat treatment, Ti-O-C chemical bonds are usually formed between PVA and TiO_2_ molecules. In contrast, during the simple composite method in our experiment, the nano-TiO_2_ was simply embedded in the PVA matrix, and no new chemical bond was formed between both materials, a phenomenon that has been reported elsewhere [24]. The results showed peaks at 1450 cm^−1^ and 2950 cm^−1^, which correspond to the C–H bending (alkane, -CH_3_, and -CH_2_) and the C–H stretching vibration from PVA, respectively.

**Figure 2:**
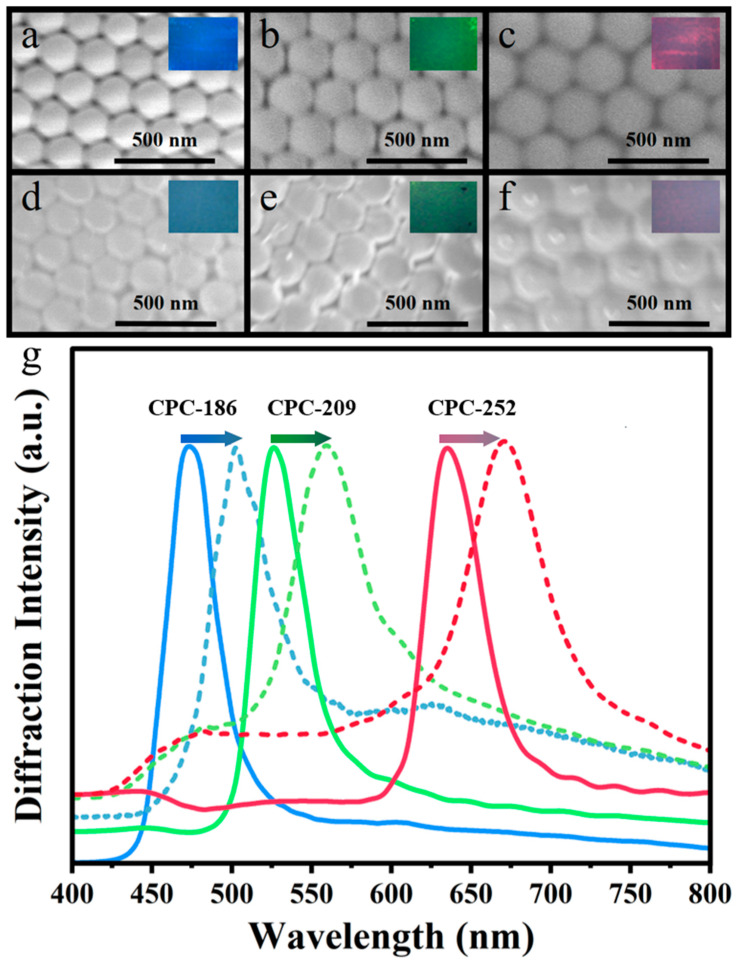
SEM of the prepared samples: (**a**) CPC-186; (**b**) CPC-209; (**c**) CPC-252; (**d**) PVA/TiO_2_/CPC-186; (**e**) PVA/TiO_2_/CPC-209; (**f**) PVA/TiO_2_/CPC-252. The insets in the upper right corner are the corresponding structural colors of the samples, respectively. (**g**) Diffraction spectra comparison of CPC: before and after infiltration of PVA/TiO_2_ (solid line: before infiltration; dashed line: after infiltration).

For the preparation of PVA/TiO_2_/CPC films with fewer defects, the preparation of CPC templates is particularly important. The CPC templates with fewer defects provide a good basis for their subsequent penetration of the PVA/TiO_2_ precursor solution into the CPC template interstitials as well as for easy unwinding from the glass slide to prepare high-quality films. As shown in Figure 2a–c, the morphology of the laboratory-synthesized polystyrene (PS) colloids (186 nm, 209 nm, and 252 nm in diameter) have uniform particle sizes. The hexagonally arranged structure consisted of the typical face-centered cubic (FCC) lattice, and thus, the photonic crystal templates prepared by the vertical deposition method were well-structured. In addition, the inset in the upper-right corner shows their single bright structural color.

In addition, the proper tilt angle of the PVA/TiO_2_ precursor when it penetrates the CPC template was also conducive to the penetration of the precursor into the interstitial space of the CPC template. From experimental experience, a tilt angle of less than 30° will result in the slow penetration of the PVA/TiO_2_ precursor into the CPC template, and the PVA/TiO_2_ precursor will lack mobility and solidify into a gel before it completely penetrates the gap of the CPC template. As the tilt angle is more than 30°, it will lead to the fast penetration of the PVA/TiO_2_ precursor into the CPC template, and the PVA/TiO_2_ precursor will not completely penetrate the gap of the CPC template. The PVA/TiO_2_ precursor solution will be slowly flowed down along the glass slide and penetrate the filling of the CPC gap; the uniformly PVA/TiO_2_ precursor-filled CPC slide was put into the oven at 37 °C for 12 h to cure into a film. The resulting high-quality PVA/TiO_2_/CPC film can be conveniently peeled off from the glass slide.

As shown in Figure 2d–f, the SEM result showed that the PVA/TiO_2_/CPC films prepared using the CPC templates remained in their ordered hexagonal pattern, and the encircling of the PVA/TiO_2_ precursor solution did not affect the periodic structure of the CPC template. Comparing the insets between Figure 2a–c and Figure 2d,e, it can be observed that the structural color of the CPC templates changed significantly after the infiltration and filling of the PVA/TiO_2_ precursor solution. The characterization of the fiber optic spectrometer revealed that the reflection peaks had undergone different degrees of red-shift phenomena, in which the reflection peak of CPC-186 was red-shifted from 471 nm to 481 nm, the reflection peak of CPC-209 was red-shifted from the original 524 nm to 560 nm, and the reflection peak of CPC-252 was shifted from 636 nm to 671 nm. The Δλ for CPC-186, CPC-209, and CPC-252 were 10 nm, 36 nm, and 35 nm, respectively. For the same reason as above, the diffraction wavelengths of the material and its average refractive index were positively correlated due to the same lattice spacing of photonic crystals. The refractive index of nano-TiO_2_ was 2.5, and the refractive index of PVA was 1.49, which is greater than the refractive index of air (1.0). After filling the CPC template gap with the precursor solution of PVA/TiO_2_, the diffraction wavelengths were red-shifted when the average refractive index increased [25,26].

**Figure 3:**
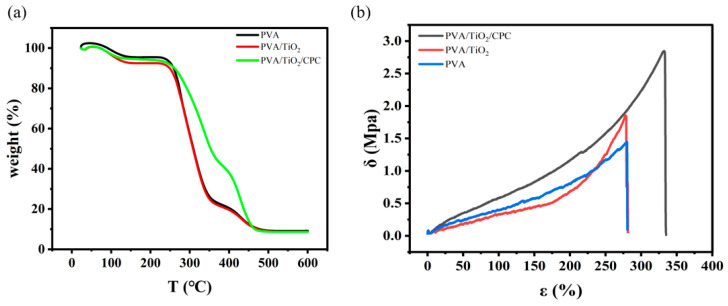
(**a**) Thermogravimetric graphs of PVA, PVA/TiO_2_, and PVA/TiO_2_/CPC films; (**b**) Strain–stress graphs of PVA, PVA/TiO_2_, and PVA/TiO_2_/CPC films.

The PVA, PVA/TiO_2,_ and PVA/TiO_2_/CPC films were thermally analyzed using a comprehensive thermal analyzer. As can be seen in Figure 3a, below 250 °C, each film showed a similar behavior. Over 250 °C, the retained weight of the three films declined differently. The weight of pure PVA and PVA/TiO_2_ films dropped dramatically, while the PVA/TiO_2_/CPC composite film dropped less than the other two films. It can also be seen that with the addition of CPC, the melting temperature of the composite film increased, which indicates that the thermal stability of the composite film was improved. The better property of the PVA/TiO_2_/CPC is due to the fact that the CPC in the composite film of PVA/TiO_2_/CPC is formed because of the hydrogen bonding force to form a dense stacking structure. In addition, we also carried out relevant tensile property tests on the films using a universal material testing machine; Young’s modulus of the films was calculated from the slope of the linear part of the stress–strain curve.

Figure 3b shows that the strain-at-break of pure PVA was 280.31% and the tensile strength was 1.43 MPa; the elongation-at-break of the composite film PVA/TiO_2_ was 277.20% and tensile strength was 1.83 MPa; the elongation-at-break of the PVA/TiO_2_/CPC was 330.53% and tensile strength was 2.84 MPa (c.f., Table 1). Pure PVA films are semi-crystalline polymers with more amorphous structural domains than ordered structural domains at room temperature, so the extension in the tensile direction and the arrangement of the lattice units of the PVA film are due to the plastic deformation of the structure. The ductility of the stress–strain curve of the pure PVA film in Figure 3b is mainly due to the amorphous chains. After the incorporation of TiO_2_ nanoparticles into PVA, the intermolecular interactions were increased, and thus, the tensile strength of the composite film was improved. While there was a slight decrease in its strain-at-break, the tensile strength of its composite film PVA/TiO_2_ was increased from 1.43 to 1.83 MPa. The elongation-at-break was decreased from the original 280.31% to 277.20% because the addition of nanoparticles increases the stiffness of the composite film while decreasing its ductility. Subsequently, combining the CPC template with the PVA/TiO_2_ composite film further increased the strain-at-break and tensile strength of the composite film, which increased the elongation-at-break from 280.31% to 330.65% and the tensile strength from 1.83 to 2.84 MPa, which significantly affected the mechanical properties of the PVA/TiO_2_ composite film. This is due to the tightly ordered arrangement of PS colloids in the CPC template so that they are uniformly dispersed into the PVA/TiO_2_ composite film. The CPC template plays the role of an active filler so that the macromolecules in the composite film form a stable structure, the interchain force increases, and the intermolecular force required to overcome the external force is greater. The addition of CPC templates increases the fracture strain of the composite film, enhances the structural rigidity of the molecular chains, and tightens the network. This improves the mechanical properties of the composite film, thus showing an obvious rise in stress–strain behavior.

### 2.2. Dark Adsorption–Photocatalytic Degradation of MB Dye by PVA/TiO_2_/CPC Film

**Figure 4:**
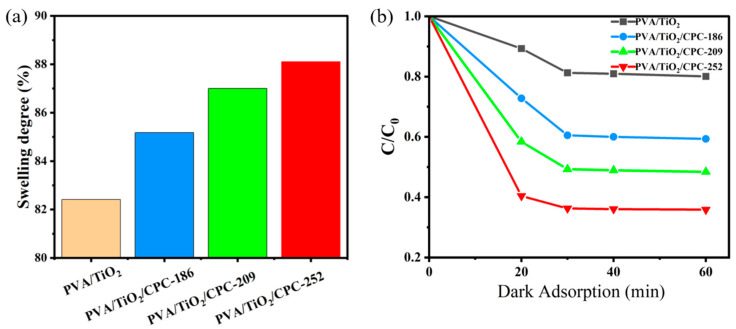
(**a**,**b**) The equilibrium swelling and rate profiles of dark adsorption of PVA/TiO_2_ and PVA/TiO_2_/CPC (186 nm, 209 nm, and 252 nm) films, respectively.

Prior to the photocatalytic degradation experiments, dark adsorption experiments were carried out on PVA/TiO_2_ and PVA/TiO_2_/CPC (186 nm, 209 nm, 252 nm) films. In the experiments, PVA/TiO_2_ and PVA/TiO_2_/CPC films were cut into the same size and placed in 10 mL of a 10 mg/L MB dye solution and then placed in a shaker at 35 °C for 60 min, then the surface of the films was wiped and weighed to calculate the equilibrium degree. The experimental results are shown in Figure 4a. The lowest equilibrium swelling rate of PVA/TiO_2_/CPC (186 nm, 209 nm, 252 nm) films was higher than that of PVA/TiO_2_ films. On one hand, the presence of PS colloids during infiltration increased the loose network structure of the films, resulting in more hydrophobic channels of the films. On the other hand, the PS colloids showed excellent binding capacity and high adsorption efficiency for certain dyes, affinity ligands, and substances. Thus, the PVA/TiO_2_/CPC films had better adsorption ability for MB compared to that of PVA/TiO_2_. As shown in Figure 4b, with the same regularity as Figure 4a, the ratio of final concentration to initial concentration after 60 min of dark adsorption of PVA/TiO_2_/CPC films was 0.59, 0.48, 0.36, respectively, while that of the PVA/TiO_2_ films was 0.80. In comparison with the PVA/TiO_2_ films, the dark adsorption removal rate of MB dye in the PVA/TiO_2_/CPC films was 2–3.2 times higher.

**Figure 5:**
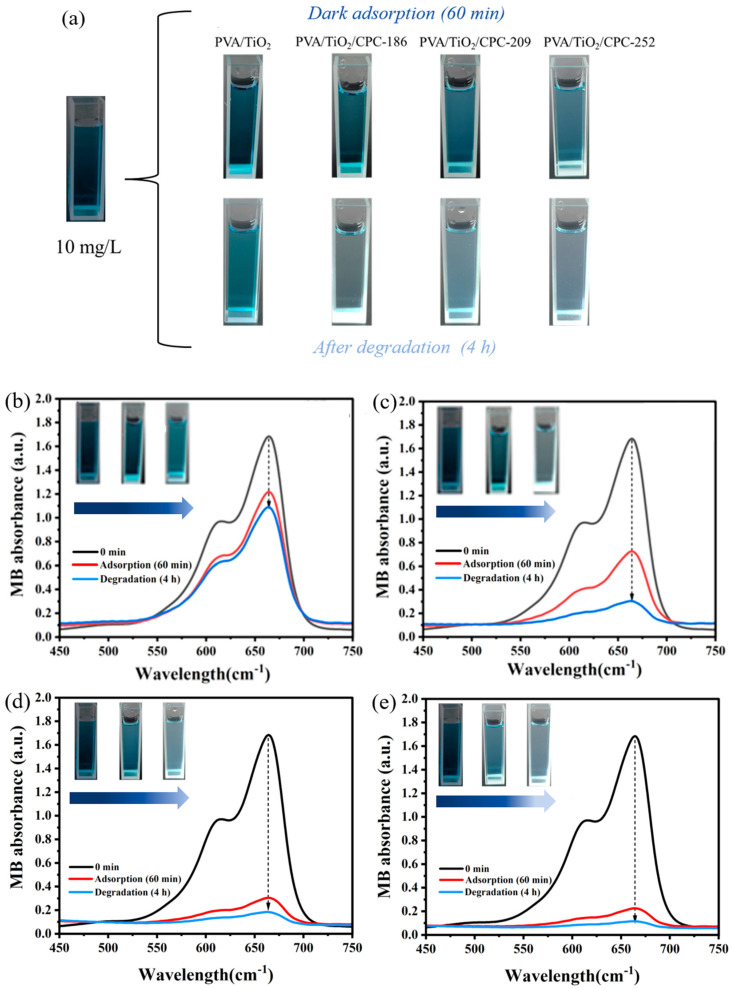
(**a**) Physical diagrams of the dark adsorption (60 min)–photocatalytic (4 h) degradation of MB dye by PVA/TiO_2_ and PVA/TiO_2_/CPC films; UV–visible absorption spectra of the degradation of MB dye by (**b**) PVA/TiO_2_, (**c**) PVA/TiO_2_/CPC-186, (**d**) PVA/TiO_2_/CPC-209, and (**e**) PVA/TiO_2_/CPC-252 films, respectively.

To investigate the difference in photocatalytic efficiency between PVA/TiO_2_ and PVA/TiO_2_/CPC films for the degradation of MB dye, we first subjected the PVA/TiO_2_ and PVA/TiO_2_/CPC films to dark adsorption in a 10 mg/L MB dye solution for 60 min in a constant temperature shaker at 35 °C and then continued to conduct the photocatalytic degradation experiments using a 10 W UV lamp. As shown in Figure 5a, the dark blue color of the 10 mg/L MB dye solution without dark treatment was gradually lightened by the dark treatment of PVA/TiO_2_ and PVA/TiO_2_/CPC composite films for 60 min. The color of the MB dye solution was the lightest after the dark treatment of the composite film PVA/TiO_2_/CPC-252. The color of the MB dye solution was consistent with the effect of the dark treatment we mentioned above. After 4 h of photocatalytic experiments, the MB dye was further degraded, and the color of its dye solution was further lightened. It can be seen that the MB dye solution changed from dark blue to light blue after photocatalysis by the PVA/TiO_2_ film, while the MB dye solution after photocatalytic degradation by the PVA/TiO_2_/CPC composite film showed different degrees of light blue, and its overall color was lighter than that of the dye after photocatalysis by the PVA/TiO_2_ film.

We used the prepared PVA/TiO_2_ and PVA/TiO_2_/CPC films to degrade the initial concentration of a 10 mg/L MB dye solution, respectively. Figure 5b–e plots the UV–visible absorption result of PVA/TiO_2_ and PVA/TiO_2_/CPC composite films that degraded the MB dye after dark adsorption (60 min)–photocatalysis (4 h), in which the absorbance of the MB dye was 1.203 after dark adsorption of the PVA/TiO_2_ film for 60 min, which reduced to 1.097 after 4 h of photocatalysis.

Meanwhile, the absorbance after dark adsorption of PVA/TiO_2_/CPC-186, PVA/TiO_2_/CPC-209, and PVA/TiO_2_/CPC-252 films was 0.725, 0.307, and 0.225, respectively, which decreased to 0.327, 0.174, and 0.111, respectively, after 4 h of photocatalysis. The 4 h photocatalytic degradation of MB by PVA/TiO_2_/CPC films was significantly better than that of PVA/TiO_2_ films, with the PVA/TiO_2_/CPC-186 film having the best photocatalytic effect, with approximately three times higher catalytic efficiency than that of PVA/TiO_2_ films. This is because of the unique structure of composite CPCs. On the one hand, its PS colloids have a negative charge, which has an excellent binding ability for the dye molecules and improves the photocatalytic efficiency of TiO_2_ nanoparticles. On the other hand, it is due to the slow photon effect on the optical property of the photonic crystals [27], which is generated by the interactions between the wave packets reflected from the photonic bandgap (PGB) and those that are non-reflective (i.e., transmissive). This can effectively promote the interaction between light and material, i.e., when the low-frequency or high-frequency edges of these slow photons overlap with the electronic band gaps (EBGs) of the photocatalysts prepared by combining photonic crystals, the photocatalytic activity can be significantly improved by increasing the effective optical path length. Briefly, the slow photon effect of photonic crystals improves the photocatalytic efficiency of TiO_2_ nanoparticles and thus enhances the photocatalytic degradation efficiency of MB in PVA/TiO_2_/CPC films.

### 2.3. Cycling Performance (Repeatability) Study

**Figure 6:**
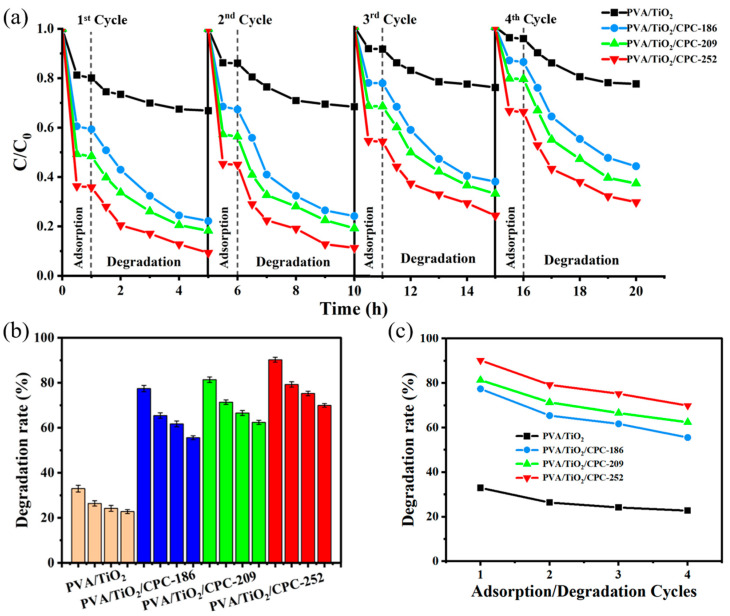
(**a**) Repeated (4 times) experiments of adsorption–photocatalytic degradation of MB by PVA/TiO_2_ and PVA/TiO_2_/CPC films; (**b**,**c**) Comparison of the removal rate of adsorption–photocatalytic degradation of MB by repeated use of PVA/TiO_2_, PVA/TiO_2_/CPC films.

The PVA/TiO_2_/CPC film catalysts were prepared by combining the PVA/TiO_2_ with the CPC template. The microstructure of the final PVA/TiO_2_/CPC thin film catalyst is a three-dimensional periodic photonic crystal. PVA/TiO_2_ and PVA/TiO_2_/CPC films were reused as reusable experimental objects. Following the steps of the adsorption–photocatalysis experiments described above, the films were reused three times to explore and compare the differences in cycling performance between the four films and the differences in adsorption–photocatalysis performance between each cycle. The degradation effect of each adsorption catalysis is shown in Figure 6a.

As shown in Figure 6b, in the first adsorption–photocatalytic degradation of MB experiment, the degradation rate of PVA/TiO_2_/CPC film catalysts for MB was approximately 77~90% in 4 h, while the degradation rate of PVA/TiO_2_ films for MB was approximately 33% in 4 h. After four cycles of adsorption–photocatalysis, the dark adsorption and photocatalytic abilities of the four films decreased (Figure 6c). The photocatalytic activity was weakened, which was attributed to the fact that with the increase in the number of dark adsorption times, the internal gel pores of the films became smaller, and the solubilization and adsorption capacity was almost saturated, making it impossible for dye molecules in solution to diffuse sufficiently. Therefore, it occupied part of the active sites on the TiO_2_ surface, which led to a decrease in the catalytic activity of the film and the weakening of the degradation ability of the dye. However, the degradation efficiency of the PVA/TiO_2_/CPC films for MB dye remained above 56~70%, which was 2.3~2.7 times higher than that of the PVA/TiO_2_ films.

The difference in the cycling performance between the PVA/TiO_2_ films and the PVA/TiO_2_/CPC (186, 209, 252 nm) films in terms of cycling performance for the same reasons is mentioned above. The cycling performance showed that the PVA/TiO_2_/CPC films prepared in this experiment could carry out the adsorption–photocatalytic degradation reaction many times and have good photocatalytic reusability performance. In addition, this experiment did not require the aid of centrifugation and other operations for the recovery of the adsorbent during the recycling process. Finally, the composite material was simple and convenient to recover and had good recyclability.

## 3. Conclusions

In this work, we used a simple composite method combining a CPC template with PVA/TiO_2_ to prepare PVA/TiO_2_/CPC film catalysts with a three-dimensional periodic structure, which consisted of PS colloids with uniform particle size and neatly arranged interconnections; the interspaces of the colloids were filled with PVA/TiO_2_ composite gels. The degradation of MB by the film catalyst showed that the degradation rate of ordinary PVA/TiO_2_ films for MB was approximately 33% within 4 h, while the degradation rate of PVA/TiO_2_/CPC film catalysts for MB was approximately 77~90% within 4 h. The adsorption–photocatalytic degradation of MB by the latter film catalyst was approximately 2.3~2.7 times higher than that of the former film catalyst. The main reasons were that the PS colloids of PVA/TiO_2_/CPC film were rich in negative charges, which have an excellent binding ability for MB-positive dye, and the PS colloids were highly reactive on the surface with high adsorption efficiency. Moreover, the slow photon effect of periodic PS colloid structures increases the optical range length of the irradiated light wave in the material, which promotes the interaction between the light and the material and is conducive to the improvement of the catalytic degradation of the photocatalytic system. Therefore, the introduction of the opal structure of photonic crystals into the photocatalytic system could effectively enhance the photocatalytic activity. In this study, it was demonstrated that the introduction of the CPC structure into the photocatalytic thin film species effectively enhanced the adsorption and photocatalytic degradation of dyes in the film.

## 4. Materials and Methods

### 4.1. Materials

Styrene, ammonium persulfate (APS), methacrylic acid (MAA), and PVA (99% hydrolyzed, DP = 1750 ± 50) were obtained from Shanghai Chemical Agent Co., Ltd., Shanghai, China. Nano-TiO_2_ (99 wt%, 20~40 nm) and methylene blue (≥95%) was purchased from Titan Scientific Co., Ltd., Shanghai, China). All other chemicals were obtained from Sinopharm Chemical Reagent Co., Ltd., Shanghai, China. Ultrapure water (18.2 MΩ·cm) was used in all experiments. All materials were used as received without further purification.

### 4.2. Synthesis of PS Monodisperse Colloids

Monodisperse PS colloids were prepared by boiling emulsifier-free polymerization [28]. At a stirring rate of 380 R/min, 5 mL of MAA and 100 mL of deionized water were added to a three-necked flask, followed by adding 40 mL of styrene (washed with 10% NaOH solution prior to use), and the mixture was heated to boiling. A total of 0.1 g of APS was added to the mixture to initiate the reaction, and the reaction was continued for 30~60 min. The flask was then moved to an ice water bath to terminate the polymerization. Finally, the latex was poured into a dialysis bag and dialyzed against deionized water for at least 7 days to remove impurities.

### 4.3. Preparation of CPC Template

A total of 0.5 mL of the above dialyzed PS latex was placed into a beaker and diluted to 50 mL with deionized water, and the glass slides were vertically placed in the diluted latex. The beaker was placed in a 65 °C oven for 48 h. After the evaporation of the water, the PS colloids self-assembled onto the glass slide due to capillary forces, and the artificial CPC templates were finally prepared after further heating at 80 °C for 8 h while the bright structure diffraction color could be observed.

### 4.4. Preparation of PVA/TiO_2_/CPC Adsorption Photocatalytic Films

**Figure 7:**
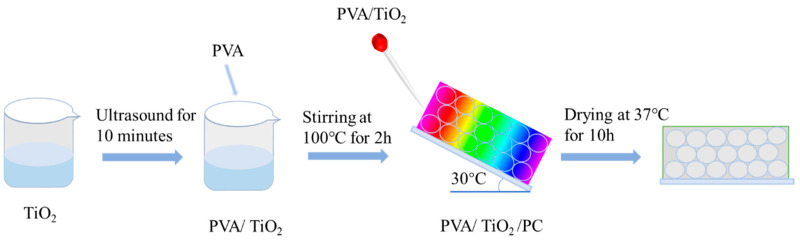
Schematic diagram of the preparation process of PVA/TiO_2_/CPC films.

A total of 6 mg of nano-TiO_2_ was placed in 10 mL of deionized water and ultrasonicated for 1 h to make the nano-TiO_2_ uniformly dispersed in the deionized water. A total of 2 g of PVA was added into the above uniformly dispersed aqueous solution of nano-TiO_2_, and then 8 g of deionized water was added into a beaker and heated and stirred at 100 °C for 2 h until the PVA was completely dissolved; the PVA/TiO_2_ precursor solution with a PVA concentration of 10 wt% was prepared. At room temperature, 0.5 mL of 25% glutaraldehyde was added to the PVA/TiO_2_ precursor solution, and to prevent excessive cross-linking of PVA, the PVA/TiO_2_ precursor solution was immediately infiltrated into the interstitial space of the CPC templates prepared in advance after stirring for 5 min at room temperature. The glass slide covered with CPC arrays on the surface was tilted at 30°, and a pipette was used to aspirate the above PVA/TiO_2_ precursor solution, which flowed down slowly along the glass sheet and penetrated to fill into the CPC gap. The uniformly filled CPC glass sheet containing the PVA/TiO_2_ precursor solution was dried in an oven at 37 °C for 12 h to form a film. The PVA/TiO_2_/CPC film was removed from the glass sheet and sealed for storage. Without penetrating the PVA/TiO_2_ precursor solution into the interstitial space of the CPC template, the PVA/TiO_2_ film was prepared under the same experimental conditions and stored in a sealed condition at room temperature. The whole preparation process of PVA/TiO_2_/CPC films is shown in Figure 7.

### 4.5. PVA/TiO_2_/CPC Dark Adsorption–Photocatalytic Degradation of MB

The 1 × 2 cm PVA/TiO_2_ film and PVA/TiO_2_/CPC film of the same size were placed in 10 mL of MB solution with an initial concentration of 10 mg/L and then dark adsorbed for 60 min in a constant temperature shaker at 35 °C in a closed and dark environment with shaking at 120 r/min. Then, the 1 × 2 cm dark adsorbed PVA/TiO_2_ film and the 1 × 2 cm dark adsorbed PVA/TiO_2_/CPC film, with a UV lamp with a power of 10 W as a light source, were dark-adsorbed in the dark adsorption process after 60 min. After 60 min of dark adsorption, the 1 × 2 cm PVA/TiO_2_ film and the PVA/TiO_2_/CPC film of the same size were subjected to photocatalytic degradation of 10 mL of MB solution with an initial concentration of 10 mg/L.

### 4.6. Characterization

**Figure 8:**
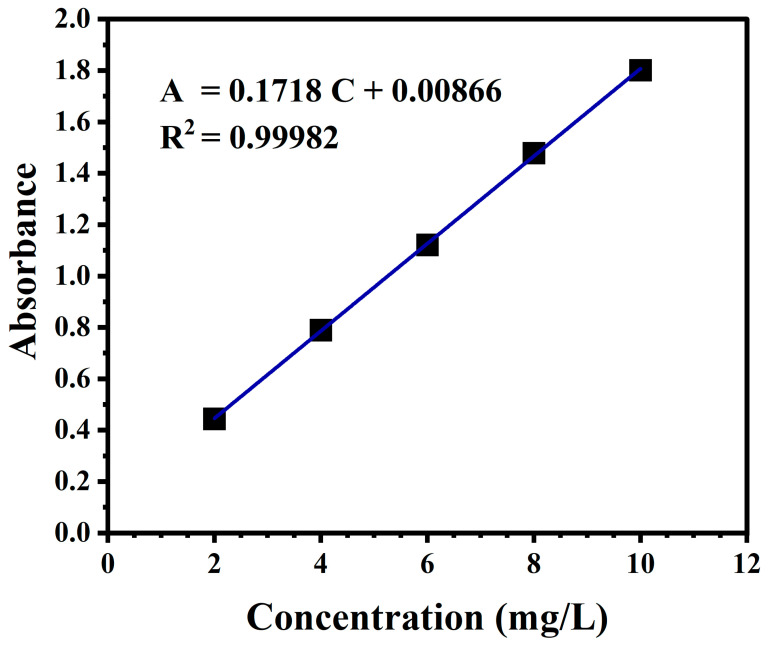
Standard curve of MB in a UV–vis experiment.

Microstructural photographs of the samples were taken using a scanning electron microscope (Hitachi, S-4800, Tokyo, Japan). Fourier transform infrared (FTIR) spectroscopy (Nicolet, iS10, Waltham, MA, USA) was used to analyze the chemical bonding between the PVA carriers and the TiO_2_ nanoparticles. An integrated thermal analyzer (TGA400 PC, NETZSCH, Selb, Germany) was used with a temperature increase rate of 10 °C and a temperature scanning range from 25 °C to 600 °C to measure the heat weight loss and energy change data of each specimen film. The diffraction spectra of the samples were determined by a fiber optic spectrometer (Ocean Optics, USB 4000-XR1-ES, Winter Park, FL, USA), with the reflectance spectra ranging between 400 and 800 nm. The absorbance of MB was tested with a UV–Vis spectrophotometer (Shimadzu, UV 2600, Tokyo, Japan) between 200 nm and 800 nm using deionized water as a blank reference sample, and the test results were recorded. Since the MB showed specific absorbance at 664 nm, whose intensity was related to the concentration of MB, the corresponding concentration can be calculated from the experimental values. The standard curve determined and used in this experiment is shown in Figure 8. Equation (1) shows the relationship between absorbance and concentration:

A = 0.1718 C + 0.00866(1)

where A is the absorbance and C is the concentration.

Optical photographs of the samples were taken by a digital camera (Canon, EOS 6D, Tokyo, Japan) with a macro lens (Tamron, 272E, Tokyo, Japan). The films were cut into standard sample sizes of 20 × 10 mm (length × width). The tensile stress δ (MPa) and tensile strain ε were tested with a Shimadzu AGS-X universal material testing machine (Kyoto, Japan) with a tensile speed of 100 mm/min and a clamp distance spacing of 30 mm at a testing temperature of 25 °C.

The tensile stress was calculated by Equation (2):

(2) $$\delta =\frac{F}{a\times b}$$

where *F* is the fracture load (N), *a* is the width of the sample (mm), and *b* is the thickness of the sample (mm).

The tensile strain was calculated by Equation (3):

(3) $$\varepsilon =\frac{L-L_{0}}{L_{0}}$$

where *L* is the entire stroke fracture or yielding (mm) and *L*_0_ is the original distance (mm).

## Figures and Tables

**Table 1:** Summary of tensile test data for PVA, PVA/TiO_2_, and PVA/TiO_2_/CPC films.

Sample	Elongation (%)	Tensile Strength (Mpa)	Young’s Modulus (Mpa)
PVA	280.31	1.43	0.44 ± 0.001
PVA/TiO_2_	277.20	1.83	0.49 ± 0.003
PVA/TiO_2_/CPC	330.65	2.84	0.72 ± 0.006

## Data Availability

The original contributions presented in the study are included in the article, further inquiries can be directed to the corresponding author.
